# Electric-field-induced alignment of electrically neutral disk-like particles: modelling and calculation

**DOI:** 10.1038/s41598-017-09180-7

**Published:** 2017-08-16

**Authors:** Rongshan Qin

**Affiliations:** School of Engineering & Innovation, The Open University, Walton Hall, Milton Keynes MK7 6AA United Kingdom

## Abstract

This work reveals a torque from electric field to electrically neutral flakes that are suspended in a higher electrical conductive matrix. The torque tends to rotate the particles toward an orientation with its long axis parallel to the electric current flow. The alignment enables the anisotropic properties of tiny particles to integrate together and generate desirable macroscale anisotropic properties. The torque was obtained from thermodynamic calculation of electric current free energy at various microstructure configurations. It is significant even when the electrical potential gradient becomes as low as 100 v/m. The changes of electrical, electroplastic and thermal properties during particles alignment were discussed.

## Introduction

Alignment of anisotropic particles toward desirable orientation has been a hot research topic for many decades^1–9^. This has two levels of implementations: The first is to align the particles to generate static anisotropic structural or functional properties. Another is to align the particles dynamically so that the anisotropic microstructure and properties can be switched promptly. A giant torque is required for achieving both fast switch and the effective fabrication.

It is known that tiny particles have their outside morphology inherited from their crystal anisotropic nature^1^. The anisotropic shape particles possess favourable anisotropic properties^2^. Randomly packed anisotropic particles, however, would wipe out the desirable anisotropic functionalities due to the statistical average. Aligned microstructures are able to integrate the microscopic anisotropy into useful macroscopic anisotropic properties. The later has many advanced engineering applications^3, 4^. For examples, aligned carbon nanotubes are being considered in nerve repairing and tissue regeneration^5^. Aligned nanostructures are desirable in electro-optical switching and memory electronics^6, 7^. Alignable graphene have been pursued for display devices^4, 8, 9^.

There are three ways to align anisotropic particles, namely the mechanical stress, electric fields and magnetic fields. Mechanical alignment is a general method that is applicable to most situations^10, 11^. But it has disadvantages including the operation complexity and possible damage to the targeted particles. The electric field and magnetic field methods are easier to manipulate than the mechanical one^4, 12^. Magnetic method makes use of the particles magnetic properties caused by magnetic response of orbit electrons. High magnetic fields are usually required in the alignment processing^13, 14^. Application of electric field to manipulate electrically neutral particles has been investigated since 1950s. Kolin pointed out that the particles with different electrical conductivities can be separated using an electric field plus an additional cross magnetic field^15^. This has been developed into a major technology for separation of different types of biological cells in blood, and has wide applications in separation of materials according to their electrical properties^16^. The mechanism is called electromagnetophoresis. Pohl proved that a non-uniform electric field could be implemented to manipulate dielectric particles^17^. The mechanism is called electrophoresis and has been used intensively in biomedical applications such as to processing bacteria and target cells^18^. It is found that direct current electric field drives multiwall carbon nanotubes move toward negative electrode and alternating current electric field can align the nanotubes in liquid solutions^12^. Our previous researches shown that electric field can drive spherical particles to move toward a wall from the inner position in liquid metal^19^ and can drive graphene nanoflakes to move toward the negative electrode^20^. It is also demonstrated recently that electric field can help to breakup droplets into fine particles^21^ and rotate crystals’ orientation^22^. The aim of the present work was to find out a mechanism to use electric field to align disc-like particles. This is to be proved in a way completely different from that of dielectrophoresis. Electric current has percolation effect, which means that electric current flows along a lowest resistance route. The hypothesis of the present work concerns a reciprocal question, i.e. can electric current help to change the microstructure and to generate a lowest resistivity route in a multiphase material?

## Results

**Figure 1:**
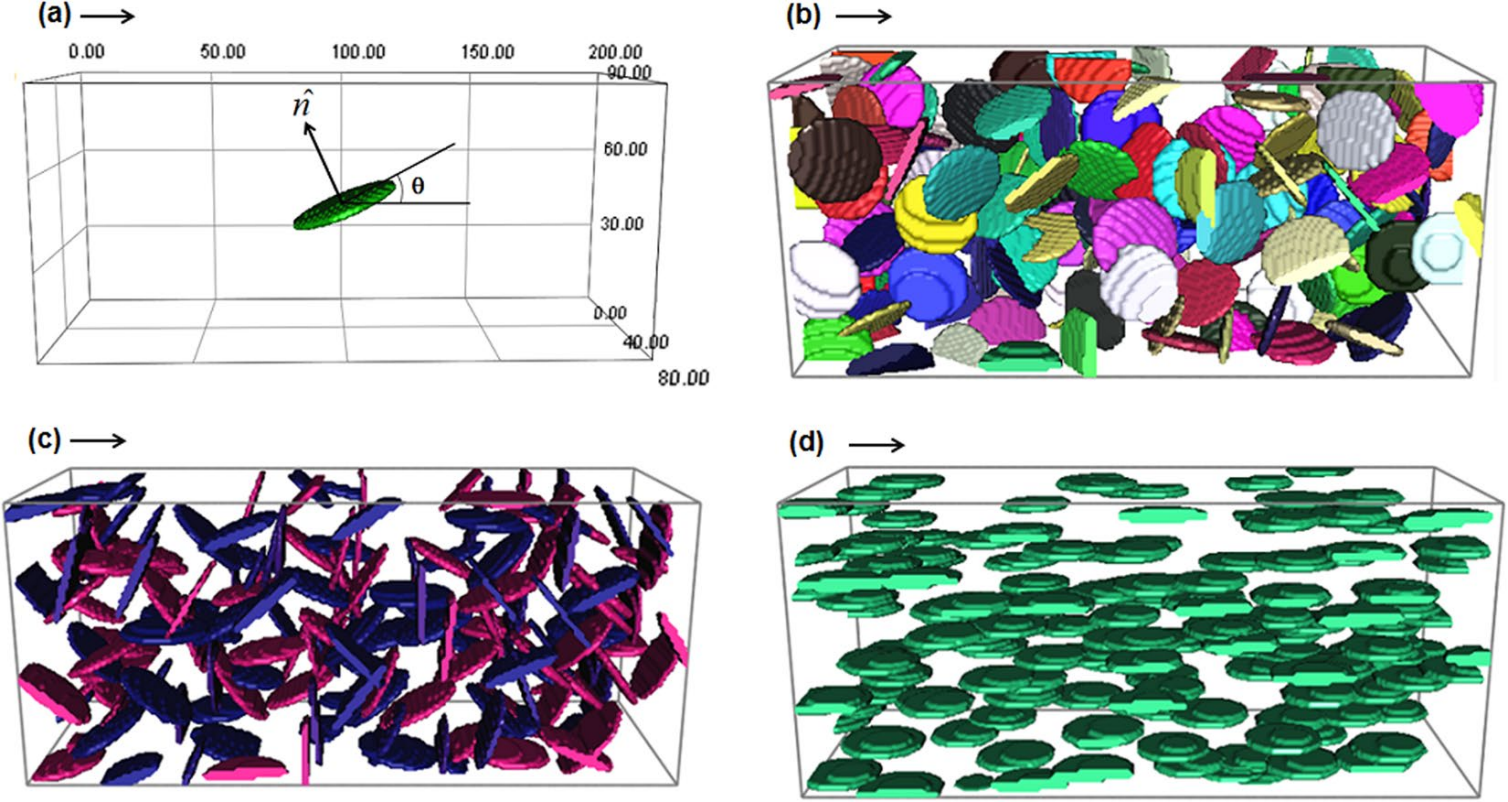
Ellipsoidal particles suspended in the matrix of dimension 200 × 90 × 80 grids. An arrow in each figure represents the direction of applied electric field. The eccentricity for all ellipsoidal particles is 0.1. (**a**) Only one particle submerged in the matrix with angle of inclination *θ* to electric field direction and the normal vector $\hat{n}$; (**b**) 173 ellipsoidal particles with random orientation; (**c**) 173 particles with some extents of preferred orientation toward vertical direction; (**d**) 173 aligned particles along current flow direction.

The resistance of a material containing anisotropic particles is dependent on the orientation of particles if the resistivity of particles is different from that of the matrix^23^. The classic linear law of mixture for materials resistivity does not suitable for the anisotropic microstructure. This work is to consider the disc-like particles suspended in a matrix. We use thin and round ellipsoidal particles to represent the disc-like particles. The system is illustrated in Fig. 1. Figure 1(a) contains only one particle, which is inclined by θ degrees from the current flow direction. $\hat{n}$ is the normal direction of the disc. The particle is perpendicular to the current flow direction when θ = 90° and is parallel when θ = 0°. To prove that electric current tends to rotate the disc-like particles from θ = 90° to θ = 0°, one needs to illustrate that the rotation reduces system free energy. In the present case, a pure rotation does not change the volume fraction of particles or matrix. The chemical free energy remains the same during rotation. The total system surface energy is also not changed due to nonexistence of coalesce and breakup. Hence the change of system free energy during particle rotation contains only the change of electric current free energy (*G*_*e*_). The second law of thermodynamics for Fig. 1(a) is hence expressed as

1 $$\frac{\delta {G}_{e}}{\delta \theta }\le 0$$

where *G*_*e*_ is the electric current free energy, which takes following expression in a static state approximation^24^.

2 $${G}_{e}=-\frac{1}{8\pi }\iint \frac{\mu (r){\vec{j}}_{\theta }(r^{\prime} )\cdot {\vec{j}}_{\theta }(r)}{|r-r^{\prime} |}drdr^{\prime} $$

where *μ*(*r*) is the local magnetic permeability at a position *r*. ${\overrightarrow{j}}_{\theta }(r)$ is the local electric current density when the particle incline angle is *θ*. Denoting the electrical conductivity and magnetic permeability of particle by *σ*_*p*_ and *μ*_*p*_ and that of matrix by *σ*_*m*_ and *μ*_*m*_ respectively, the different values of *θ* cause different spatial distributions of electrical and magnetic properties in the system. This will again cause different electric current density distributions in the system, which leads to different values of electric current free energy according to Eq. (2).

The analytical solution for current density distribution in Fig. 1 is not possible to obtain by solving Maxwell equations. Numerical calculations have been carried out instead. A logistic frame with 200 × 90 × 80 cubic lattice has been implemented in the present work. In construction of microstructure, an nucleus was introduced in the centre of the logistic frame and then to grow with a velocity $\vec{v}={\hat{v}}_{x}+0.1{\hat{v}}_{y}+{\hat{v}}_{z}$. The anisotropic growth rate enables the introduced ellipsoidal geometry with eccentricity of 0.1. The dimension of velocity was 1 lattice distance per time step. The growth in Fig. 1(a) was stopped when the volume of ellipsoidal particle reaches 4500 grids. This gives the volume fraction of particle around 0.3%. A 3 × 3 Euler angles rotation matrix is then applied to the particle so that the desirable value *θ* is generated.

**Figure 2:**
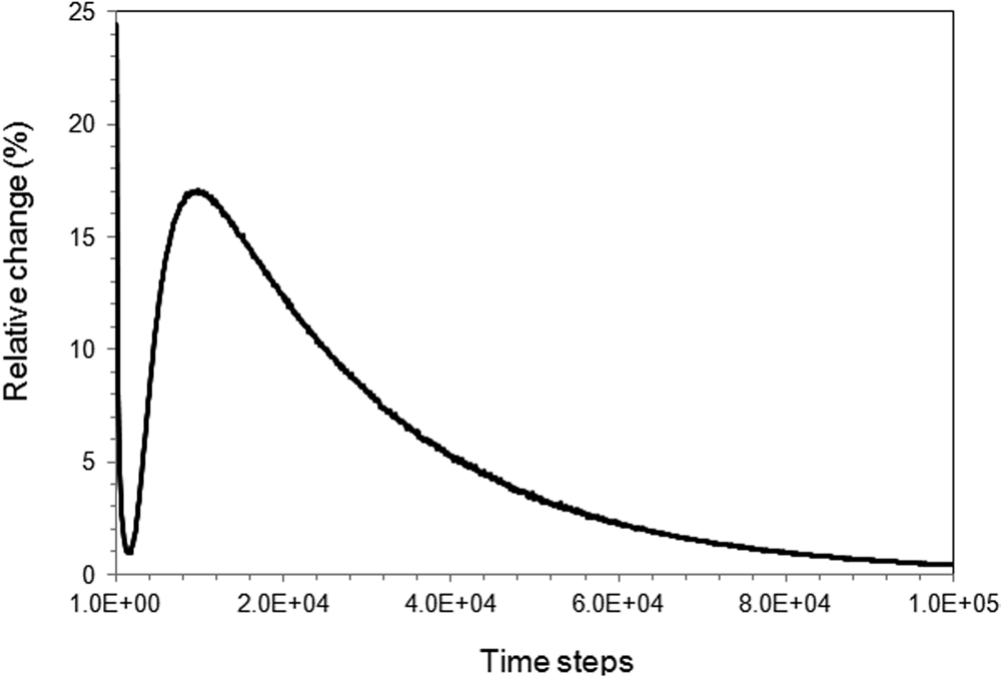
The convergence of relaxation method in the calculation of current density distribution.

One applies *V* = 20 *volts* electrical potential along the longest dimension of cuboid logistic frame. Without losing generality but for the convenience of experimental validation, one uses *σ*_*m*_ = 9.17 × 10^6^ *S*·*m* ^−1^ and *μ*_*m*_ = 300 *μ*_0_, where *μ*_0_ = 1.26 × 10^−6^ *N*·*A* ^−2^ is the vacuum permeability^25–28^. These parameters are taken from the corresponding properties of ferrite iron. The properties of ellipsoidal particle are assumed to be *σ*_*p*_ = 1.22 × 10^6^ *S*·*m* ^−1^ and *μ*_*p*_ = 30 *μ*_0_^25–28^. These are selected after the references of Fe_3_C cementite particles. The electrical resistivity of interface is assumed to have the average value between that of the adjacent phases. This is equivalent to a Stephan sharp interface approximation so that the resistance is equivalent to that of the serial connection between two resistors. The distribution of electric current density is calculated using Kirchhoff’s circuit laws. In the calculation, non-dimensionalization was performed to the parameters using $\tilde{j}=j/{j}_{0}=j[{\rm{\Delta }}{x}_{0}/({V}_{0}{\sigma }_{0})]$, $\tilde{\sigma }=\sigma /{\sigma }_{0}$, $\tilde{x}=x/{x}_{0}$, $\tilde{V}=V/{V}_{0}$ and $\tilde{T}=T/{T}_{0}=T[{\rm{\Delta }}{x}^{2}\rho c/{V}_{0}^{2}{\sigma }_{0}{\rm{\Delta }}t]$, where Δ*x*_0_ is the grid distance of logistic frame, *V*_0_ = 1*volt*, *σ*_0_ = 10^7^ *S·m* ^−1^, Δ*t* is the electric current load duration, *ρ* and *c* are the mass density and specific heat of local phase, respectively. A relaxation method has been applied to calculate the current density distribution. The initial condition was set as $\tilde{V}=20$ at the left boundary of frame and ${\tilde{V}}_{ijk}=0$ at all the rest grids with grid coordinates (*i*, *j*, *k*) in three-dimensional system. The direction of the applied electric field is illustrated in Fig. 1. During calculation, the values of relativity change was calculated by $(\sum _{ijk}{\tilde{V}}_{ijk}^{t+1}-\sum _{ijk}{\tilde{V}}_{ijk}^{t})/\sum _{ijk}{\tilde{V}}_{ijk}^{t}\cdot 100 \%$. Its value has been recorded, as shown in Fig. 2 as a typical trend. The steady state distribution of electric current is considered to be achieved when the relativity change of electrical potential across the logistic frame is less than 10^−5%^. The electric current free energy was calculated according to the steady state electric current. One thing to be pointed out is that all the calculations are based on static approximation. The non-equilibrium effect, such as skin effect has not been taken into account.

**Figure 3:**
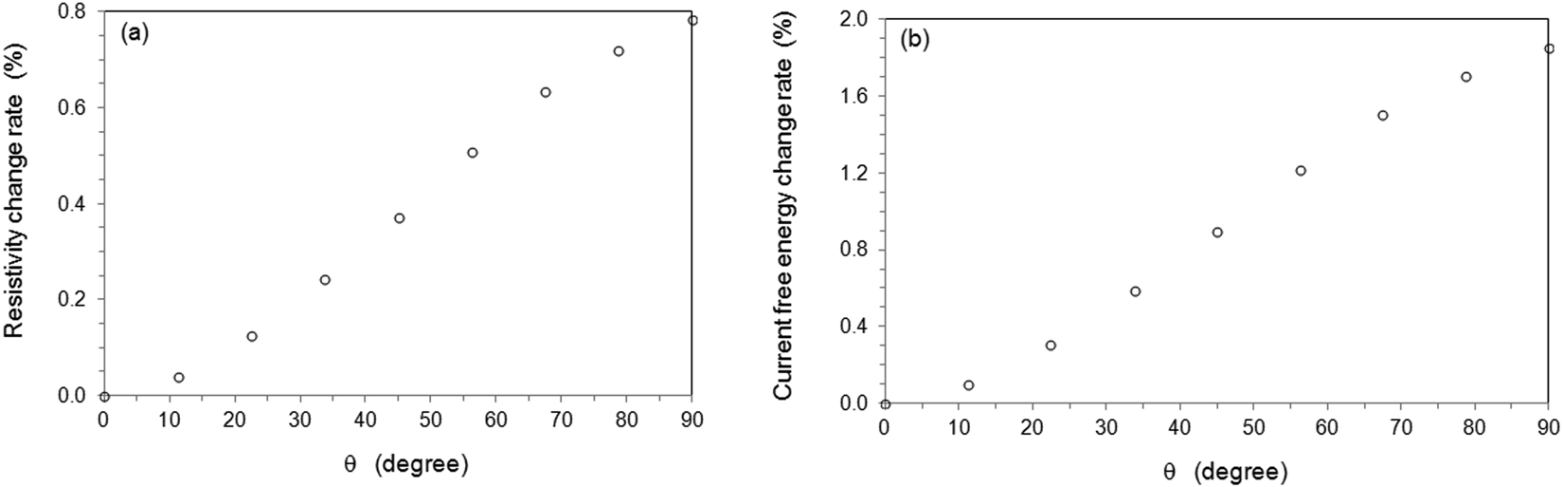
Property changes during the rotation of ellipsoidal particle. (**a**) Change of apparent electrical resistivity of system; (**b**) Change of apparent electric current free energy.

The numerical calculation is able to provide electrical resistance of the system for various microstructural configurations. Figure 3(a) shows the *θ*-dependent resistance for Fig. 1(a). When the decline angle of particle changes from 0 to 90°, the apparent resistivity $({\tilde{R}}_{\theta }-{\tilde{R}}_{\theta =0})/{\tilde{R}}_{\theta =0}$ $=\,({\tilde{\rho }}_{\theta }-{\tilde{\rho }}_{\theta =0})/{\tilde{\rho }}_{\theta =0}$ increases by 0.8%. where ${\tilde{\rho }}_{\theta }$ is the average resistivity for *θ*. Giving the volume fraction of particle is 0.3 only, the change of electrical resistivity during the rotation of the anisotropic particle is significant. An anisotropic particle with higher eccentricity ratio will contribute even more. The result shows that the orientation of suspended particles to the electrical properties is considerable. This is different from the classic linear law of mixture^23^. In fact, the orientation of anisotropic particles affects many other properties such as flow permeability^29^, optical^30^, and others^31, 32^.

Figure 3(b) shows the change of apparent electric current free energy $({\tilde{G}}_{e}^{\theta }-{G}_{e}^{\theta =0})/{\tilde{G}}_{e}^{\theta =0}$ when the incline angle reduces from 90° to 0°. It shows that the electric current free energy decreases monotonically when the ellipsoid particle rotates toward the alignment along electric current direction. Eq. (1) is satisfied for the rotation. The disc-like particle will be aligned toward the direction parallel to that of the electric current in order to reduce electric current free energy.

**Figure 4:**
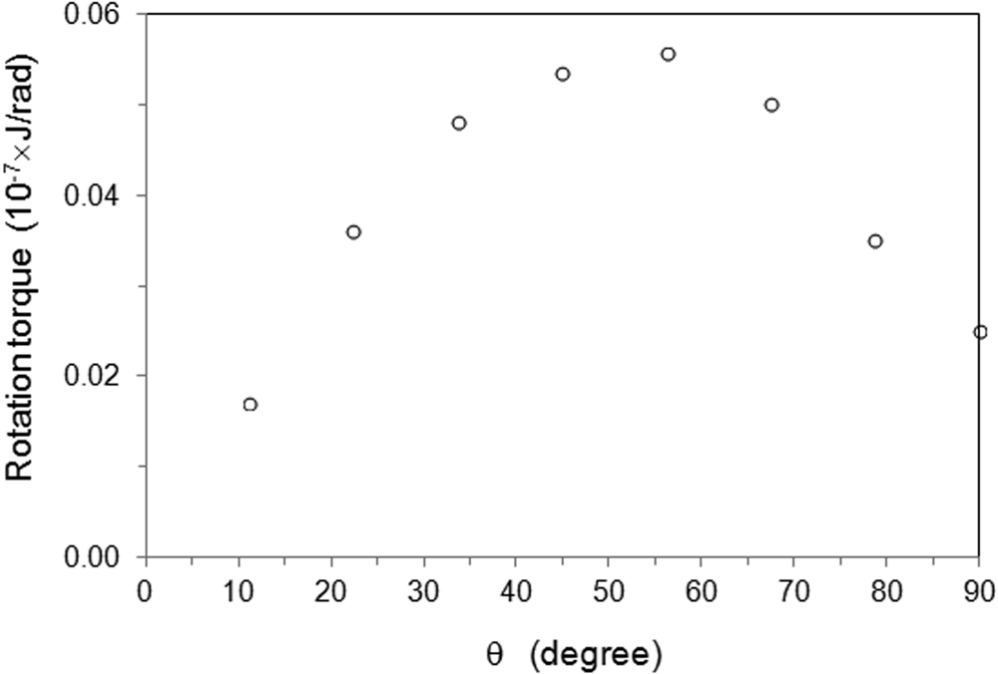
The electric-current-induced torque is dependent on the incline angle.

As is known, the reduction of free energy can be used to do work. It has −*G*_*e*_ = *τθ* for rotation, where τ is torque. The electric-current-induced torque for rotation has been calculated according to change of electric current free energy during particle rotation. The results for Δ*x* = 10^−8^ *m* are plotted in Fig. 4. The value of torque is in a magnitude of 10^−9^ J/rad. To illustrate the contribution of this torque to the rotation of a disc-like particle, one uses the density of cementite of *d* = 7730.14 *kg*/*m* ^3^ and get the moment of inertia as $I=\sum _{i}{m}_{i}{r}_{i}^{2}$ = 2.88 × 10^−31^ kg·m^2^. This gives an angular acceleration without consideration of viscosity in a magnitude of 10^21^ rad/s^2^, which is very large. The angular acceleration is proportional to the square of voltage potential gradient. Even if the electrical potential reduces from the present value of 10^7^ v/m to 100 v/m, the torque can still generate an angular acceleration of 10^11^ rad/s^2^, which is still significantly large. This is in agreement with experimental observations^33^.

For a system containing many disc-like particles, as shown in Fig. 1(b–d), the electric current free energy is −2.21 × 10^−7^ Joules, −1.92 × 10^−7^ Joules and −2.55 × 10^−7^ Joules, respectively. The state with fully aligned particles along electric current direction, Fig. 1(d), has the lowest electric current free energy. The random orientated particles, Fig. 1(b), have an electric current free energy between Fig. 1(c and d). Figure 1(c) possesses some orders along a direction perpendicular to the current flow direction, which has higher electric current free energy than both Fig. 1(b and d). Figure 1(b–d) contains 173 disc-like particles with total particle volume fraction 12.21%. In another group of calculations, a system containing 100 disc-like identical orientation particles of total particle volume fraction 30% has electric current free energy reduced from −0.47 × 10^−7^ Joules at θ = 90° to −0.81 × 10^−7^ Joules at θ = 45° and eventually to −1.52 × 10^−7^ Joules at θ = 0°. Electric current tends to rotate the disc-like particles along a direction parallel to the electric current flow.

It should be pointed out that rotating a disc-like particle to a direction parallel to the current flow direction does not mean the normal direction (see in Figure 1(a)) of all particles to point toward the same direction. In order to align the normal direction of plates to desirable direction, subsequent electric current processes are required. For example, rotating the sample around a vertical axis by 90° and then subjected to the electric potential would help to further align the particles’ normal direction and to achieve a state of Fig. 1(d).

**Figure 5:**
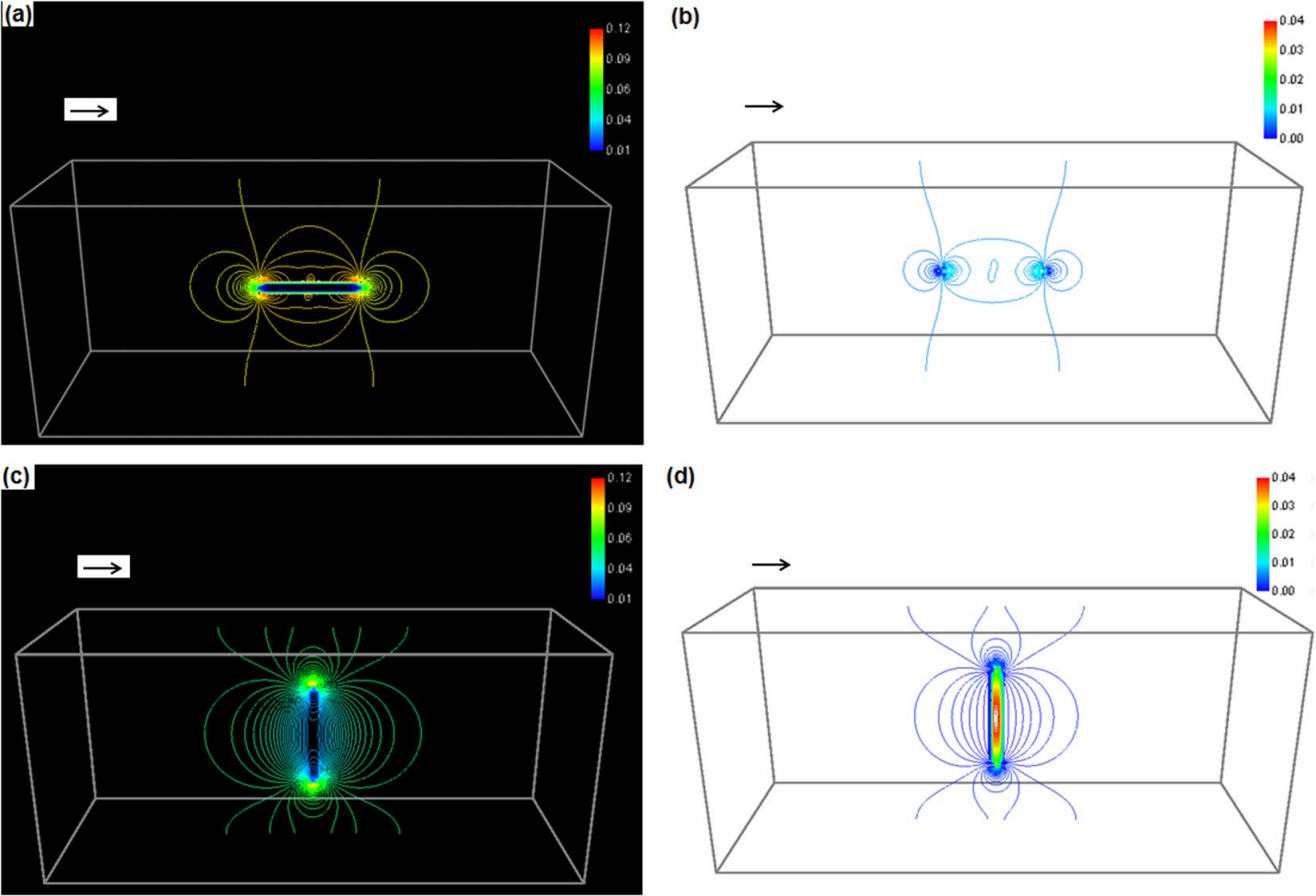
Distribution of apparent electric current density ((**a**) and (**c**)) and apparent Ohm-heat-induced temperature ((**b**) and (**d**)), where (**a**) and (**b**) are with incline angle θ = 0°; (**c**) and (**d**) are with incline angle θ = 90°. The arrows in the figure represent the applied electric field.

Apart from the change of electrical conductivity during the rotation and alignment of anisotropic particles, other properties such as electroplascity, electromigration and Ohm heat distributions change as well. Figure 5(a) and (c) plot the apparent current density distribution for incline angles at 0 and 90°, respectively. The current density inside the particle is lower than that of the matrix due to its high electrical resistivity and is dependent on the orientation of particles. Electroplasticity and electromigration effects are dependent on the local electric current density. Figure 5(b) and (d) are apparent Ohm-heat-induced temperature distribution when the incline angles are 0 and 90°, respectively. The apparent temperature inside the particle is higher than that of the matrix when θ = 90° but is lower than that of the matrix when θ = 0°. The distribution changes smoothly during the rotation. The scale of temperature is in a dimension of ${V}_{0}^{2}{\sigma }_{0}{\rm{\Delta }}t/({\rm{\Delta }}{x}^{2}\rho c)$.

## Discussion

The derivation of the rotation torque in the present work is based on thermodynamic consideration and calculation. This is different from that of deriving electromagnetophoresis^15^ and dielectrophoresis^18^. Electromagnetophoresis was derived by considering the change of force on a spherical particle when it moves across an electric current and a homogeneous magnetic field which is perpendicular to the electric current^15, 16^. The dielectrophoresis was derived by replacing a portion of spherical medium with material of different permittivity, which is equivalent to introduce a dipole moment into an electric field^17, 18^. Both theories were based on dynamic considerations and have not included the anisotropic particles. This work does not include a magnetic field, and hence is different from electromagnetophoresis. The dipole moment in dielectrophoresis was calculated according to electrostatic field distribution of an infinite large system containing a spherical particle. The derivation of dielectrophoresis of spherical particles in finite system is complex^34^. The dielectrophoresis of anisotropic particles in a finite system is difficult to derive by the dynamic method.

**Figure 6:**
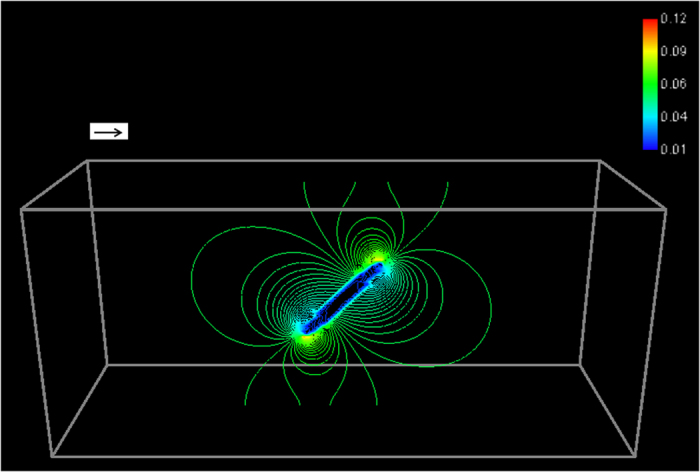
The electric current density distribution when the particle’s incline angle is 45°. The arrow represents the direction of applied electric field.

The electric current distribution pattern illustrated in Fig. 5(a) is similar to that of a dipole moment superimposed to a uniform electric current field. However, the pattern in Fig. 5(c) is more similar to a dipole to present in vertical direction. It is difficult to image that a horizontal uniform electric field could induce polarization of a particle in vertical direction. Figure 6 presents the electric current density distribution of one disc-like particle with incline angle of 45°. The current distribution is sensitive to the particle orientation. The geometry and orientation alter the current distribution significantly, which again affect the electric current free energy. The lowest free energy position is the most stable position, which is a state the longest axis of disc-like particles parallel to the current flow direction.

This work investigated the electric-current-induced alignment of suspended anisotropic particles in fluid medium. In fact, the effect has been observed in many other solid-state experiments in low-carbon automotive steels^35^ and high silicon electrical steels^36^. The difference between the alignment in solid and liquid medium is that the former is via reconstruction and the latter is by rotation.

## Methods

### Software

The three-dimensional visual analysis of the computational data was generated using MatVisual software.

### Data availability statement

The datasets generated during and/or analysed during the current study are available from the corresponding author on reasonable request.

## References

[1] Ringe E, Van Duyne RP, Marks LD (2011). Wulff construction for alloy nanoparticles. Nano Lett..

[2] Bonaccorso F, Sun Z, Hasan T, Ferrari AC (2010). Graphene photonics and optoelectronics. Nat. Photonics..

[3] Sacanna S (2013). Shaping colloids for self-assembly. Nat. Commun..

[4] Lin F (2017). Orientation control of graphene flakes by magnetic field: broad device applications of macroscopically aligned graphene. Adv. Mater..

[5] Cunha C, Panseri S, Antonini S (2011). Emerging nanotechnology approaches in tissue engineering for peripheral nerve regeneration. Nanomed. Nanotech. Biol. Med..

[6] Shen TZ, Hong SH, Song JK (2014). Electro-optical switching of graphene oxide liquid crystals with an extremely large Kerr coefficient. Nat. Mater..

[7] Hu ZJ, Tian MW, Nysten B, Jonas AM (2009). Regular arrays of highly ordered ferroelectric polymer nanostructures for non-volatile low-voltage memories. Nat. Mater..

[8] Comiskey B, Albert JD, Yoshizawa H, Jacobson J (1998). An electrophoretic ink for all-printed reflective electronic displays. Nat..

[9] Ferrand H (2016). Magnetic assembly of transparent and conducting graphene-based functional composites. Nat. Commun..

[10] Haggenmueller R, Gommans HH, Rinzler AG, Fischer JE, Winey KI (2000). Aligned single-wall carbon nanotubes in composites by melt processing methods. Chem. Phys. Lett..

[11] Thostenson ET, Chou TW (2002). Aligned multi-walled carbon nanotube-reinforced composites: processing and mechanical characterization. J. Phys. D: Appl. Phys..

[12] Oliva-Aviles AI, Aviles F, Sosa V, Oliva AI, Gamboa F (2012). Dynamics of carbon nanotube alignment by electric fields. Nanotechnol..

[13] Kimura T (2002). Polymer composites of carbon nanotubes aligned by a magnetic field. Adv. Mater..

[14] Smith BW (2000). Structural anisotropy of magnetically aligned single wall carbon nanotube films. Appl. Phys. Lett..

[15] Kolin A (1953). An electromagnetokinetic phenomenon involving migration of neutral particles. Science.

[16] Leenov D, Kolin A (1954). Theory of electromagnetophoresis. I. Magnetohydrodynamic forces experienced by spherical and symmetrically oriented cylindrical particles. J. Chem. Phys..

[17] Pohl HA (1958). Some effects of nonuniform fields on dielectrics. J. App. Phys..

[18] Pethig R (2010). Review article-dielectrophoresis: status of the theory, technology, and applications. Biomicrofluidics..

[19] Zhang XF, Qin RS (2015). Controlled motion of electrically neutral microparticles by pulsed direct current. Sci. Rep..

[20] Zhang XF, Qin RS (2015). Preparation of surface coatings on a conductive substrate by controlled motion of graphene nanoflakes in a liquid medium. Appl. Surf. Sci..

[21] Qin RS (2017). Using electric current to surpass the microstructure breakup limit. Sci. Rep..

[22] Rahnama A, Qin RS (2017). Room temperature texturing of austenite/ferrite steel by electropulsing. Sci. Rep..

[23] Fan Z (1995). A new approach to the electrical resistivity of two-phase composites. Acta Metall. Mater..

[24] Qin RS, Bhowmik A (2015). Computational thermodynamics in electric current metallurgy. Mater. Sci. Technol..

[25] Bohnenkamp U, Sandström R, Grimvall G (2002). Electrical resistivity of steels and face-centered-cubic iron. J. Appl. Phys..

[26] Zhang P, Wang XB, Wang W, Lei X, Yang H (2015). Iron carbide and nitride via a flexible route: synthesis, structure and magnetic properties. RSC Adv..

[27] Thompson SM, Tanner BK (1993). The magnetic-properties of pearlitic steels as a function of carbon content. J. Magn. Magn. Mater..

[28] Hao XJ (2008). Off-line measurement of decarburization of steels using a multifrequency electromagnetic sensor. Scr. Mater..

[29] DeRocher JP, Gettelfinger BT, Wang JS, Nuxoll EE, Cussler EL (2005). Barrier membranes with different sizes of aligned flakes. J. Membrane Sci..

[30] Mobini E, Rahimzadegan A, Alaee R, Rockstuhl C (2017). Optical alignment of oval graphene flakes. Optics Lett..

[31] Tie WW (2013). Dynamic electro-optic response of graphene/graphitic flakes in nematic liquid crystals. Optics Express.

[32] Chen JY, Lim B, Lee EP, Xia YN (2009). Shape-controlled synthesis of platinum nanocrystals for catalytic and electrocatalytic applications. Nano Today.

[33] Kosc TZ, Marshall KL, Jacobs SD, Lambropoulos JC, Faris SM (2002). Electric-field-induced motion of polymer cholesteric liquid-crystal flakes in a moderately conductive fluid. Appl. Optics.

[34] Lo YJ, Lei U (2009). Quasistatic force and torque on a spherical particle under generalized dielectrophoresis in the vicinity of walls. Appl. Phys. Lett..

[35] Rahnama A, Qin RS (2015). Electropulse-induced microstructural evolution in a ferritic–pearlitic 0.14% C steel. Scr. Mater..

[36] Hu GL, Shek C, Zhu YH, Tang GY, Qing X (2010). Effect of electropulsing on recrystallization of fe-3%si alloy strip. Mater. Trans..

